# Does “Rural” Always Mean the Same? Macrosocial Determinants of Rural Populations’ Health in Poland

**DOI:** 10.3390/ijerph17020397

**Published:** 2020-01-07

**Authors:** Paulina Ucieklak-Jeż, Agnieszka Bem

**Affiliations:** 1 Department of Economics and Finance, Czestochowa, Jan Dlugosz University in Czestochowa, 42-200 Częstochowa, Poland; 2 Department of Corporate and Public Finance, Wrocław University of Economics and Business, 53-345 Wrocła, Poland; agnieszka.bem@ue.wroc.pl

**Keywords:** rural health inequalities, social determinants of health, concentration analysis, HHI

## Abstract

Rural areas, as well as urban ones, are not homogeneous in terms of social and economic conditions. Those surrounding large urban centers (suburban rural areas) act different roles than those located in remote areas. This study aims to measure the level of inequalities in social determinants of health (SDH) between two categories of rural areas. We pose the following research hypotheses: (hypothesis H1) rural areas in Poland are relatively homogenous in the context of SDH and (hypothesis H2) SDH affects life expectancies of rural residents. Based on data covering all rural territories, we found that rural areas in Poland are homogenous in SDH. We also find important determinants of health rooted in a demographic structure—the feminization index and a ratio of the working-age population. On the other hand, we cannot confirm the influence of commonly used SDH-GDP and unemployment rate.

## 1. Introduction

Social determinants of health (SDH) comprise of economic and social conditions, as well as their distribution in the particular populations. The most commonly cited are income, education, unemployment and job security, employment and working conditions, early childhood development, food insecurity, housing, social exclusion, social safety network, health services, aboriginal status, gender, race, and disability [1]. SDH covers, therefore, all factors determining the condition of a human organism, both physical and psychological. In other words they describe non-medical conditions that influence populations’ health SDH [2,3,4,5,6]. These factors, combined or individually, can exert a favorable or unfavorable impact on the health of individuals, as well as the entire population. The catalogue of potential SDH is virtually unrestricted, so in previous studies, authors employ a wide range of factors (Table 1), and the choice of analyzed factors is partly determined by the characteristics of the research group (children, immigrants, and older people). On the other hand, some authors propose to group SDH into clusters having the same origin or characteristics. Orpana and Lemyre introduce a division into three broad categories: material/structural, behavioral/lifestyle, and psychosocial mechanisms [7]. Bethune and colleagues propose to group SDH into the following categories: the structural factors having a distal character, which is rooted in socioeconomics and politics (like income, education, and gender) and the intermediary determinants regarded as proximal ones that flow out of the structural determinants (like stressors or social isolation) [8].

Hence, the place of residence, through a specific combination of SDH, influences individual’s state of health [33,34,35,36,37,38]. Usually, the dissimilarity between an urban and rural residence are the centre of attention, as the result of clear differences in SDH. Cities offer a positively denser social and institutional network what contributes to better health in several ways [39,40]. Rural areas, as territories of lower urbanization, are exposed to several negative consequences—residents of rural areas are particularly vulnerable to social deprivation [16]—there are, on average, less educated that generate lower earnings, more often perform physical work, and are exposed to harmful factors related to agricultural production. Moreover, villagers have usually worse access to health care benefits [41,42]. Village residents rarely benefit from regular doctor visits [43,44] including preventive services [45] and tend to have weaker access to emergency services [46,47], which are usually located in urban areas. These differences in SDH contribute to health inequalities expressed by dissimilar life expectancies or healthy life expectancies [33].

Life expectancy (LE) is the most commonly used indicator that provides an overall assessment of a population’s health [48]. LE is usually estimated for a newborn and a person at the age of 65 years, which bases on the assumption that this age draws a line of increasing demand for health benefits. LE weakness is that it does not take into account the quality of life associated with the burden of diseases. Factors such as mortality and morbidity are incorporated into other indicators, like disability-free life expectancy (DFLE) or healthy life expectancy (HLY). Both DFLE or HLY are calculated using the Sullivan method based on the data of incidence, prevalence, and disability distributions for selected diseases [49,50,51].

Previous research in this area focuses primarily on the differences in health state between urban and rural populations, explained by SDH. Generally, LE and HLY, as well as DFLE, is lower for rural residents [52]. However, rural areas should not be perceived as homogeneous in terms of social and economic conditions. The level of socio-economic development of rural regions varies—the most developed rural areas are located in the vicinity of large and medium-sized cities [53]. Rural areas surrounding large urban centers (suburban rural areas) act different roles than those located in remote areas. These differences include almost all factors defined as SDH: employment and income structure, education level, and access to social infrastructure. The areas around large cities are more densely populated and usually serve as a reservoir of the labor force for large cities. The proximity of highly urbanized areas often can be a source of a better financial situation of surrounding municipalities so that they can offer public services at a higher level. According to that, many important research questions arise: do rural areas differ in the context of SDH? Do these differences, if they exist, affect, and how, the state of health of the rural residents?

## 2. Methodology and Data

This study aimed to measure the level of inequalities in SDH. Using measures of inequality, we analyze the degree of inequalities for selected SDH inside macroregions and between two categories of rural territories: those located in the direct proximity of large cities (PLM) and areas located away from large urban centers (PLW). The research hypotheses were as follows:

Hypothesis H1: rural areas in Poland are relatively homogenous in the context of SDH;

Hypothesis H2: SDH affects life expectancies of rural residents.

In the construction of the H1 hypothesis, we assumed that rural areas in Poland, despite various functions and characteristics, are homogenous in the context of the selected SDH. That does not preclude a situation that the study identifies regions characterized by higher and smaller differentiation. We measured the level of inequality using two indicators: the Herfindahl-Hirschman Index (HHI) and the GINI coefficient (GINI).

We also assumed that selected SDH affects rural populations’ state of health expressed by LE for a newborn (hypothesis H2). We are forced to use LE for a newborn because other health states’ indicators are unavailable for analyzed territorial division units.

### 2.1. Reseach Sample

Poland is located in Central Europe—from the North Poland borders is Russia and Lithuania, from the east is Belarus and Ukraine, and from the south is Slovakia and the Czech Republic, with Germany on the West. Poland has a population of 38.1 million and an area of 312,685 km^2^ (2018). It is the largest country among the new Member States adopted after 2004. The Human Development Index for Poland is 0.865 (2018), which gives it the 33rd place in the World. Health services are financed within compulsory public health insurance. Of the population 40% lives in rural areas characterized as “a thinly populated area”. The rural inhabitants are, on average, less educated (higher education has 12% of people, while in cities 28%) and achieve lower income (21.9% of the rural inhabitants are in the first income quintile, comparing to 10.8% of urban inhabitants, while in the fifth quintile, 8.2% of rural inhabitants and 21.2% of urban ones). 

The research sample comprises of 73 rural sub-regions classified into 16 macro-regions (voivodeships; Lower Silesian, Kuyavian-Pomeranian, Lubelskie, Lubuskie, Łódź, Lesser Poland, Masovian, Opole, Podkarpackie, Podlaskie, Pomeranian, Silesian, Świętokrzyskie, Warmian-Masurian, Wielkopolska, and Zachodniopomorskie). Additionally, all rural sub-regions are split into two categories: sub-regions surrounding big urban centers (suburban areas; PLM) and the rest of rural areas (PLW). The research sample covers all the country’s territory. We estimated a level of inequality for seven macro-regions: southern (PL2), northwestern (PL4), southwestern (PL5), northern (PL6), central (PL7), eastern (PL8), and the Masovian voivodeship (PL9).

### 2.2. Variables

Based on previous studies (Table 1) and data availability, we selected four categories of SDH: demography, labor market, education, communities’ economic situation, and households’ access to infrastructure (Table 2) [54]. Data comes from the research “Statistical information system of rural areas”, which bases on a census and official sources and covers the years 2006–2016. Thereupon the data cover the whole rural population. The methodology of the identification and the division of subregions complies with the OECD regional typology, which is based on the degree of urbanization [55,56,57,58].

### 2.3. Statistical Analysis

In the first step of research, we employed basic descriptive statistics to describe selected variables in terms of their distributions. We analyzed, for each variable, the percentage of the average value in the maximum value, kurtosis, skewness, and variability. The share of average values in the maximum average values for the variable allows determining the disproportion of variables. It means that the disproportion of the variable is high when this indicator takes lower values. 

Then we proposed the Herfindahl–Hirschman Index (HHI), which allows assessing a level of concentration, and as a consequence, the level of inequality, for analyzed variables. The index is calculated as the sum of the squares of the shares of each variable in the overall sum of variables (characteristics), according to the following formula (1):

(1) $$HHI=\sum_{i=1}^{n}{\left(\frac{n_{i}}{n}\right)}^{2}=\sum_{i=1}^{n}\omega_{i}^{2},$$

where:

$\omega_{i}$—the proportion of a percentage of a variable for *i* sub-region to a percentage of a variable in all sub-regions.

The interpretation basis on the following ranges of values:

- HHI < 1500—lack of concentration;
- 1500 < HHI < 2500—moderate level of concentration;
- HHI > 2500—highly level of concentration [59,60,61].

Moreover, we employed the Gini coefficient as a measure of concentration (inequality).

(2) $$GIN\left(x\right)=\frac{\sum_{i=1}^{n}\left(2i-n-1\right)x_{i}}{n^{2}\bar{x}},$$

where:

*x_i_*—*i*-unit value of analyzed phenomenon,
$\bar{x}$—arithmetic mean,
*i*—position in a series,
*n*—sample size.

Gini coefficient takes values in the range [0; 1] but is often expressed as a percentage [62,63,64,65].

In the second step, we estimated a single-equation econometric model using the Ordinary Least Squares (OLS) method, where the dependent variable is a life expectancy for a newborn (LE). We wanted to measure the strength and direction of the relationship between selected SDH and LE. Since the study did not focus on health-related factors, we used the LE for a newborn. We could not use indicators like HLE or DFLE due to the data structure.

The model should be linear towards its parameters, and the number of observations must be higher than the number of parameters. There should be no linear dependencies among the exogenous variables. In the final stage of this empirical study, the estimated econometric models were verified. The number of observations only allows estimating the simple regression. Moreover, this small number of observations does not authorize the proper identification of the shape of the dependency. The use of more extensive equations requires an increase in the number of degrees of freedom. That is why we were forced to use the methods of spatial econometrics. Gretl supports calculations.

## 3. Results

Table 3 presents the percentage of the average value in the maximum value and Table 4—the descriptive statistics for all analyzed variables. First, all rural areas were relatively homogenous in the context of feminization (FM), the working-age population (WAP), the old-age dependency ratio (ODR), and community’s income per capita (FR). We can also observe high homogeneity in the case of selected public services like access to pre-school education (PPE) or the water supply system (WSS). In the case of the rest of the variables, the level of homogeneity was lower. We could interpret it as relative homogeneity. 

In financial terms, there were more considerable differences (in favor of municipalities around large cities) in the own resources of municipalities (OSR), targeted public grants (TG), and general subvention from the state budget (GS). However, this did not significantly change the overall financial situation of municipalities—per capita indicators only slightly favored suburban municipalities, which by nature are more densely populated. The location near a large urban centre was also associated with higher GDP, but, on the other hand, higher levels of recorded unemployment (UR). That does not necessarily mean lower unemployment levels in remote rural areas, where unemployment sometimes has a hidden character. Regardless of that, we could not clearly state that rural areas surrounding large cities were in a more favorable situation in the context of SDH. 

Table 4 presents basic descriptive statistics for macroregions (PL2–PL9) and two types of rural areas—surrounding big urban centers (PLM) and more remote (PLW). The kurtosis helps to detect “tailedness” of empirical distributions, while skewness informs about the “long tiles”. The Gini coefficient measures a level of inequality.

For all variables, we can observe essential differences in kurtosis. For the same variable, the distributions took a different shape: from platykurtotic (negative values) to leptokurtic (positive values). That means that the variables’ values were, depending on a macroregion, focused more or less around the centre point—only in a few cases, the distribution was similar to a normal one. The skewness behaves similarly—for the same variable, we observed both distributions with a left tail (negative values) and a right tail (positive values).

On the other hand, the Gini coefficient did not show a high level of variation inside macroregions that it was comparable between them. The level of inequalities was generally low (FM, ODR, ER2, UR, WAP, GDP, OSR, FR, PPE, WSS, and SS) or moderate. 

Except for demographic variables (FM and ODR) and WAP, which strongly related to the demographic situation, all variables show medium or high variability, indicating tiny differences in demographic structure, not only inside sub-regions but also between them. In particular, we did not see differences in volatility between the two categories of rural areas (PLW and PLM; Table 4).

Rural areas, however, had a very diverse economic structure. The characteristics that most differed in these areas were employment structure, expressed by the ratios of the population employed in industry, services, and financial sector (ER2, ER3, and ER4), as well as unemployment (UR). As a consequence, rural areas surrounding large city centers were more homogenous in terms of generated GDP. Rural areas, on the other hand, were more diverse in terms of access to infrastructure (WSS, SS, and GSS). The mean value of the volatility was lowest for WSS, PPE, and FR. For these characteristics, the average variability coefficient was lower than 10%, indicating that those variables were not statistically significant. 

The combination of measures presented above allows an elaborate assessment of each variable. For example, in the case of access to a gas supply system (GSS), we could observe that it was characterized by a moderate level of inequality but higher in the case of remote rural areas. In four macroregions distributions were platykurtic while in three, leptokurtic. In the case of more remote areas, values were more concentrated around the focal point of the distribution than in the case of PLM areas. The same pattern was visible in the case of the variation coefficient. In almost all regions distributions had a right tail. We could conclude that the access to a gas supply system was moderately unequal in macroregions and PLW areas were more diversified in this context then PLM areas. 

In the next step, we estimated the concentration level for selected SDH, using the nominal data due to the determination of the structure indicators. The HHI level generally shows a low level of variable concentration, describing the selected SDH (Table 5). However, there were some regions that were characterized by high or moderate concentration of SDH. Rural areas in three regions: southwestern, central, and the Masovian (metropolitan) voivodeship were characterized by moderate or even very high level of inequality in terms of SDH. These areas were characterized by the existence of a very fast growing economic centre (Warsaw, Wrocław, or Łódź), which were surrounded by relatively low developed territories. 

What is interesting, if we compared two types of rural areas (PLW and PLM), we did not observe the significant inequality, although concentration coefficients for urban areas were slightly higher, still indicating small inequalities. 

Additionally, we estimated the value of the Gini coefficients, separately for two categories of rural territories—those surrounding big city centers (PLM) and more remote areas (PLW). For the PLM group, it was equal to 0.19, while in the PLW group it was 0.20. That proves that, first, the level of SDH inequalities in those two types of rural areas were virtually the same, and, secondly, this level of inequality was low. These findings allowed us to adopt the hypothesis H1, suggesting that rural areas are homogenous in terms of SDH. This homogeneity was expressed by, not only, average values, but also by the same level of inequality (HHI and Gini).

In order to verify the H2 hypothesis, we estimated the following econometric model, separately for men and women:

(3) $$LE=a_{0}+a_{1}\;\times \;\mathrm{FM}+a_{2}\;\times \;\mathrm{ODR}\;+\;a_{3}\;\times \;\mathrm{ER}1\;+\;a_{4}\;\times \;\mathrm{ER}2+a_{5}\;\times \;\mathrm{ER}3\;+\;a_{6}\;\times \;\mathrm{UR}\;+\;a_{7}\;\times \;\;\mathrm{WAP}+a_{8}\;\times \;\mathrm{GDPr}\;+\;a_{9}\;\times \;\mathrm{OSR}\;+\;a_{10}\;\times \;\mathrm{TG}\;+\;a_{11}\;\times \;\mathrm{GS}\;+\;a_{12}\;\times \;\mathrm{FR}\;+\;a_{13}\;\times \;\mathrm{PPE}\;+\;\;a_{14}\;\times \;\mathrm{WSS}\;+\;a_{15}\;\times \;\mathrm{SS}\;+\;a_{15}\times \mathrm{GSS},$$

Estimation results for the male population are presented in Table 6 and Table 7. All variables were highly statistically significant. The model indicates five variables that positively correlated with LE for men population: FM, GS, PPE, SS, and GSS.

We checked the normality of the distribution of the random component. H0 hypothesis: the random component has a normal distribution. Test statistics: Chi-square (2) = 1.34456, *p* = 0.510543.

A more feminized environment seems to encourage the prolongation of men’s lives. When the feminization index rises by 1%; the life expectancy will extend by 0.17%, provided that the value of other variables does not change. The model also indicates variables that correlate negatively with LE for men. These are ODR, WAP, OSR, TG, and WSS. In the case of OSR, TG, and WSS variables, the interpretation of the obtained results was somewhat tricky. We could only interpret the relationship between the situation on the labor market and life expectancies—the model suggests that the lowering resource of the working population affected life expectancy negatively. If the percentage of the working-age population lowers by 1%, the LE for men extends by 0.23%, if the value of other variables is constant. The interpretation of other coefficients was analogical. However, their values were very low, so the potential to stimulate, or destimulate, LE was rather weak.

**Figure 1:**
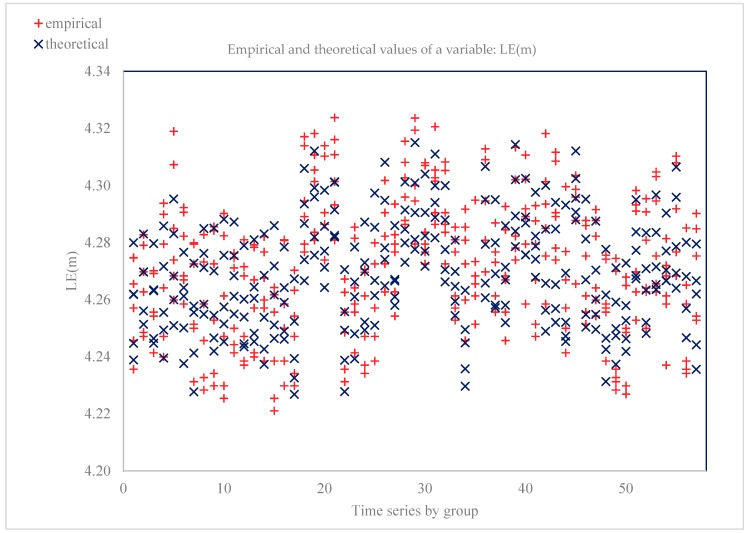
Empirical and aligned variable values (dependent variable LE(m) = 0).

In the estimated model, the coefficient of determination was 0.84, which confirms that the equation explains 84% of the variability of the explained. Hence, the model was well fit to the data. The standard error of the rests, that is, the root of rests’ variance describes the behavior of the explained variable. For model 1, it was equal to 1004, which means that the estimated LE(m) = 0 will change, on average, by ±1004 units. H0 hypothesis: experiential distribution has a normal distribution. Asymptotic test statistics: z = −0.197331 with *p* = 0.843568. Figure 1 illustrates the empirical and aligned values of the model 1 variable. 

A similar model was estimated for life expectancy for women LE(f) (Table 8 and Table 9). We generally observed the same relationship as in the case of model 1, except two variables that had an opposite direction: FM and ODR. The higher share of the female population seemed to limit LE, while the higher value of dependency ration stimulated LE positively, although this influence was minimal. It could also be concluded that the strength of this linear relationship between analyzed variables studied and women’s life expectancy was lower than for the male population (all coefficients in the model 2 had lower values than in the model 1). We could search for the source of this relationship in observation, that along with age, the share of women in the population increased as an effect of overall higher life expectancy for all women’s age groups (81.8 years, comparing to 74.4 for men, in 2017).

**Figure 2:**
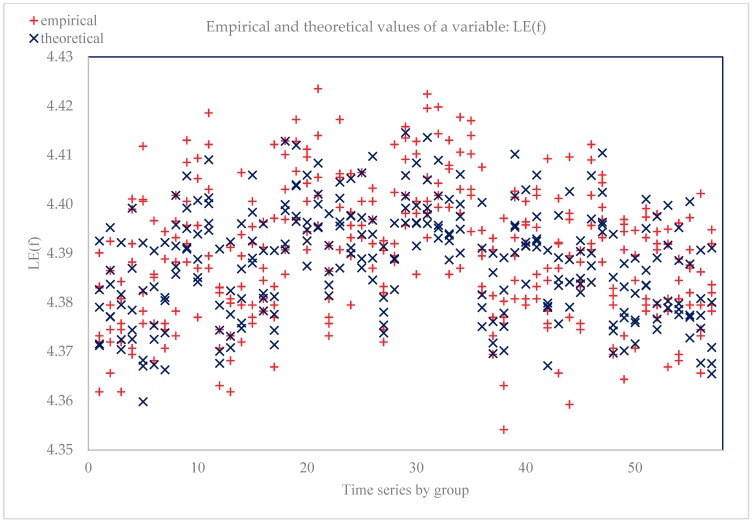
Empirical and aligned variable values (dependent variable LE(f) = 0).

The determination coefficient for model 2 took a value of 0.81, which shows that the equation explains 81% of the variability of the dependent variable. The model very well fitted the empirical data. The standard error was 1.010, which means that the estimated LE 0 will change, on average, by ±1010 units. H0 hypothesis: experiential distribution has a normal distribution. Doornik–Hansen test (1994) transformed skewness and kurtosis: chi-square(2) = 0.572684 with *p* = 0.751006. Figure 2 shows the empirical and aligned values of the model 2 dependent variable. 

To summarize, estimated models allowed us to adopt the H2 hypothesis, but there were some limitations. Although both models explained a large part of the variability of the dependent variable (R-squared) however, the strength of their influence on life expectancy was relatively small (small values of coefficient). This pattern was particularly visible in the model estimated for the female population. The exception was two variables: the feminization index and working-age population ration, but this effect was important (in terms of strength) only for the male population.

## 4. Discussion and Conclusions

The study was inspired by the observed diversity of rural areas in terms of functions, economic development’ level, and access to social and technical infrastructure. So, we posed a question whether this diversity would be visible at the level of SDH. We could conclude that in terms of analyzed SDH, rural areas were quite homogenous. Even if observed, differences acted in different directions (like GDP and unemployment rate), so we could not accept the assumption that some areas were more favorable than others.

This pattern also applies to the problem of inequality in SDH. We have not confirmed the existence of inequalities in SDH between rural areas located around large cities and those more remote. On the other hand, the study shows that there were three macro-regions in Poland, which were characterized by moderate or even high inequality in demographic structure, labor market, economic development, or access to technical infrastructure. This is an essential signal for stakeholders responsible for cohesion policy and public health.

Estimated econometric models also confirmed the impact of selected SDH on the life expectancy of women and men, although in most cases this relationship was quite weak, especially for the female population. However, it should be stressed out that in this study employed variables that have so far been rarely used in research in this area.

In the case of the male population, two factors drew our attention primarily. The impact of the feminization factor on the expected life expectancy of women and men was an exciting output of the presented study. Previous studies identify gender as an essential determinant of health state or life expectancy. We proved that the highly feminized environment positively affected men’s life expectancy but negatively influenced the life expectancy of women. This conclusion requires intense further research. High, or low, rate of participation of women in society can be linked, indirectly or directly, to other factors studied earlier, such as friendly neighborhood [9,12,17] psycho-social environment [9,15], social support [22,27], marital status [23,31], or even culture [8,16]. The second interesting determinant was the percentage of the working-age population. This factor, importantly, and negatively, affected male life expectancy.

We were a little bit surprised that both models did not confirm the relationship between GDP and life expectancies. We have expected to bear out the existence of this link based on previous studies that identify income as a major SDH [2,8,9,11,13,16,19,22,28] and, additionally, research that positively verified the impact of GDP or national wealth [17,23]. 

We also expected to confirm the impact of unemployment on life expectancy [13,15,19,22,31]. Furthermore, none of the models confirmed this relationship though many researchers consider it downright obvious.

The SDH problem is still a topical subject of research around the world. We hope that the presented study provides new evidence in this area and would be a voice in the discussion of the future shape of social policy. This paper contributes to science in several ways. Previous research generally focuses on the differences between rural and urban areas. We tried to assess the homogeneity of rural areas in the context of SDH.

Additionally, we provided new evidence in the area of SDH—we showed the role of demographic structure (especially feminization index). At the same time, we did not confirm the importance of factors rooted in previous research. We also proposed to use the Herfindahl–Hirschman Index (HHI) as a tool allowing us to measure the inequalities in SDH.

## Figures and Tables

**Table 1:** Social determinants of health in selected previous studies.

Authors	Determinants
Chang, C.D.	Health care system, poverty, housing insecurity and homelessness, education, and immigration policies and laws [2]
Spencer, N.	Income, health behaviors, birth weight, psycho-social environment, education, nutrition and diet, and environmental exposures [9]
Bissell, P.	Social deprivation [10]
Davis, S.L., and Chapa, D.W.	Socioeconomic position, social class, gender, ethnicity, education, occupation, and income [11]
Bethune, R., Absher, N., Obiagwu, M., Qarmout, T., Steeves, M., Yaghoubi, M., … and Farag, M.	Income, location, age, education, gender, culture, and volunteering [8]
Monette, L.E., Rourke, S.B., Gibson, K., Bekele, T.M., Tucker, R., Greene, S., … and Bacon, J.	Socio-demographic characteristics: age, gender, sexual orientation, education, employed, annual income; health risk behaviors: harmful alcohol use, harmful drug use, significant depression, general health; housing-related characteristics: region, unstable housing, history of incarceration, history of homelessness, experienced housing discrimination, perceived reasons of discrimination, costs of rent, and satisfaction with neighborhood and location [12]
Kolahdooz, F., Nader, F., Yi, K.J., and Sharma, S.	Income, employment, housing, and education [13]
Graham, H., and White, P.C.L.	Economic systems, built environment, living and working conditions, lifestyles, and environmental conditions [14]
Bryant, P.H., Hess, A., and Bowen, P.G.	Economic stability (e.g., poverty or employment status), education (e.g., high school graduation rates or safe school environments that encourage learning), social and community context (e.g., discrimination and equity perceptions or family structure), health and health care (e.g., access to primary care and health services), and neighborhood and built environment (e.g., quality housing or access to healthy foods) [15]
Dixon, J., and Welch, N.	Socioeconomic status (income, education, occupation), race and the indigenous health differential, environmental factors, risk-taking behaviors, physical and cultural access to services, and psychosocial factors [16]
Viner, R.M., Ozer, E.M., Denny, S., Marmot, M., Resnick, M., Fatusi, A., and Currie, C.	Structural determinants: systems and opportunities: national wealth and income inequality, education, war and conflict, sex and ethnic inequalities. proximal determinants: the circumstances of daily life: school environment, families, neighborhoods, health behaviors, and peers [17]
Mackenbach, J.P., Bopp, M., Deboosere, P., Kovacs, K., Leinsalu, M., Martikainen, P., … and de Gelder, R.	Poverty, education, and behavioral risks (smoking, alcohol abuse, and obesity) [18]
Hanibuchi, T., Nakaya, T., and Honjo, K.	Income, education, occupation, and identification (with a societal class) [19]
Zhang, A., Padilla, Y.C. and Kim, Y.	Social climate (frequency of bullying and forms of bullying), sociodemographic context (mother’s age, mother’s race, mother’s education, mother’s relationship status, church attendance), housing conditions, and control variables (age, gender, and diagnosed health condition) [20]
Kjellsson, S.	Accumulated occupational class position (unskilled manual workers, skilled manual workers, assistant non-manual employees, intermediate non-manual employees, higher level non-manual employees, self-employed, and farmers), years in work, age, and education [21]
Orpana, H.M., and Lemyre, L.	Stressors (personal, marital, children, family health, job strain, neighborhood, financial, and life events) and income [7]
Kosteniuk, J.G., and Dickinson, H.D.	Household income, education level, employment status, domestic status, retirement status, age, gender, stressor index, control, self-esteem, social support, and social involvement [22]
Maskileyson, D.	Years since migration, age, education, marital status, gender, family size, health insurance, region, income, ethnicity, Gini coefficient, and gross national income [23]
Brønnum-Hansen, H., and Juel, K.	Smoking, gender, education [24]
Bauer, G.F., Huber, C.A., Jenny, G.J., Müller, F., and Hämmig, O.	Socio-economic status, physical working conditions (exposure to physical disturbances, physical strain), psychosocial working conditions (job insecurity, monotonous work, handling simultaneous tasks, and handling new tasks) [25]
Chandola, T., Ferrie, J., Sacker, A., and Marmot, M.	Age, employment grade, retirement status, and gender [26]
Matthews, S., Manor, O., and Power, C.	Health-related behavior, family structure/social support, working characteristics, material circumstances, education and health, and gender [27]
Singh-Manoux, A., Adler, N.E., and Marmot, M.G.	Subjective social status, occupation, education, personal income, household income, and household wealth [28]
Brønnum-Hansen, H., Andersen, O., Kjøller, M., and Rasmussen, N.K.	Education and gender [29]
Kimbro, R.T., Brzostek, S., Goldman, N., and Rodríguez, G.	Education, race/ethnicity and nativity, age, and sex [30]
Theodossiou, I., and Zangelidis, A.	Demographics (age, sex, family status, and marital status), socio-economics (income, professional status, deprivation, education, employment status, industry sector), lifestyles (smoking, coffee and tea drinking, consumption of meat, fishes and veggies, alcohol consumption, regular exercises, clubs’ membership, and private health insurance), and country of residence [31]
Hämmig, O., Gutzwiller, F., and Kawachi, I.	Sex, age, educational level, nationality, civil status, occupational position, activity rate, lifestyle factors (frequent drinking, smoking, poor diet, physical inactivity, and BMI), physical work factors (lifting/carrying heavy loads, poor posture, uniform arm/hand movement, computer work, physical exposures at work, psychosocial work factors (long workdays, monotonous work, night/weekend/shift work, “people work”, job insecurity, influence at work, and social support [32]

**Table 2:** Selected socio-economic determinants of health [59].

Variable		Description	Category
FM	Feminization ratio	Females per 100 males	Demography
ODR	Old-age dependency rate	Population in the post-production age to 100 people of working age	Demography
ER 1	Employment rate in agriculture	The percentage of the population aged 15–64 working in agriculture, forestry, hunting and fishing	Labor market
ER 2	Employment rate in the industry	The percentage of the population aged 15–64 working in industry and construction	Labor market
ER 3	Employment rate in services	The percentage of the population aged 15–64 working in the trade, repairing of vehicles, transport and the warehouse industry, accommodation and catering, and information and communication	Labor market
ER 4	Employment rate financial sector	The percentage of the population aged 15–64 working in the financial and insurance sector and real estate market service	Labor market
UR	Unemployment rate	The number of unemployed people as a percentage of the labor force	Labor market
WAP	Working-age population	The percentage of the working-age population	Labor market
GDP	Gross domestic product	Gross domestic product (current prices) in PLN	Economic
OSR	Own-sources	Community’s own-source revenue (local taxes and dues)	Economic
TG	Grants	Community’s targeted grants from the state budget	Economic
GS	General subvention	General subvention from the state budget (based on financial condition)	Economic
FR	Income	Community’s income per capita in PLN	Economic
PPE	Pre-primary education	The number of children aged 3–6 attending pre-primary education per 1000 children aged 3–6	Education
WSS	Water supply	The percentage of people using the water supply system	Infrastructure
SS	Sewage system	The percentage of the population using the sewage system	Infrastructure
GSS	Gas supply	The percentage of the population using a gas supply system	Infrastructure

**Table 3:** Percentage of the average values in maximum values by macro-regions and types of rural areas (%).

Variables	PL2	PL4	PL5	PL6	PL7	PL8	PL9	PLW	PLM
FM	97.53	97.28	96.46	97.15	97.41	96.77	96.13	95.00	96.42
ODR	77.94	91.18	89.95	83.47	91.29	80.11	86.90	71.86	79.89
ER1	46.41	63.67	55.27	69.46	49.01	60.10	52.99	31.06	40.31
ER2	35.56	49.55	65.15	63.81	57.51	61.15	64.60	31.65	45.84
ER3	50.97	27.75	41.56	47.8	56.77	67.57	34.12	38.23	35.09
ER4	39.38	39.72	74.42	46.05	58.92	59.53	35.89	36.84	32.16
UR	49.42	62.97	78.22	61.49	58.43	62.20	50.39	38.35	52.23
WAP	97.58	98.36	98.8	97.92	97.78	97.85	98.31	97.22	97.23
GDP	67.59	69.01	63.75	70.02	80.27	74.09	60.17	47.95	69.79
OSR	45.39	45.47	53.45	40.05	50.60	67.68	45.52	35.73	48.15
TG	40.98	57.57	63.58	47.17	74.40	61.61	77.96	43.72	52.12
GS	42.55	53.47	66.96	43.05	72.21	61.86	73.12	39.23	50.6
FR	85.24	85.13	81.78	87.63	80.22	92.73	83.64	80.35	84.18
PPE	90.87	78.64	84.31	77.82	93.58	86.14	82.43	74.02	80.20
WSS	82.58	96.85	92.82	94.78	95.76	87.06	93.44	86.4	91.93
SS	58.45	78.59	76.67	70.17	61.20	52.93	60.06	54.78	68.61
GSS	55.79	36.59	49.32	32.04	38.75	39.37	29.86	23.63	40.66

**Table 4:** Basic descriptive characteristics by macro-regions and types or rural areas.

Variables	Statistic Measures	PL2	PL4	PL5	PL6	PL7	PL8	PL9	PLW	PLM
FM	kurtosis	0.56	0.38	4.54	0.49	3.19	0.33	−0.22	−0.22	−0.75
	Gini coefficient	0.01	0.01	0.01	0.01	0	0.01	0.01	0.01	0.01
	skewness	−0.59	0.12	2.04	0.53	1.72	−0.51	0.77	0.36	0.13
	coefficient or variation	2%	1%	2%	1%	1%	2%	2%	2%	2%
ODR	kurtosis	0.92	−0.3	−1.82	−0.45	−0.68	1.16	2.02	−0.24	−0.94
	Gini coefficient	0.07	0.03	0.04	0.06	−0.01	0.06	0.04	0.08	0.08
	skewness	0.4	−0.39	0.32	0.13	0.13	0.98	1.26	0.42	−0.03
	coefficient or variation	13%	6%	7%	10%	6%	11%	7%	15%	14%
ER1	kurtosis	0.05	−0.96	4.44	-0.86	4.89	−1.48	2.35	1.23	−0.76
	Gini coefficient	0.31	0.2	0.18	0.19	−0.11	0.24	0.22	0.39	0.39
	skewness	0.81	0.03	2.01	−0.32	2.15	0.12	1.42	1.3	0.77
	coefficient or variation	56%	35%	38%	33%	48%	43%	42%	73%	72%
ER2	kurtosis	7.31	0.65	−1.57	−0.45	−0.57	−1.78	−0.91	4.42	3.62
	Gini coefficient	0.27	0.25	0.19	0.19	−0.02	0.22	0.18	0.27	0.2
	skewness	2.52	1.06	0.57	−0.42	0.88	0.33	0.81	1.69	1.72
	coefficient or variation	60%	47%	35%	35%	42%	39%	33%	53%	39%
ER3	kurtosis	3.67	8.36	5.11	1.9	−0.53	−0.69	5.48	6.89	2.77
	Gini coefficient	0.17	0.36	0.29	0.23	−0.06	0.16	0.36	0.18	0.33
	skewness	1.95	2.79	2.21	1.22	1.15	0.32	2.3	1.81	1.78
	coefficient or variation	36%	90%	64%	44%	43%	29%	82%	36%	67%
ER4	kurtosis	4.95	1.18	−2	1.3	1.5	−0.06	6.47	3.23	4.53
	Gini coefficient	0.27	0.34	0.14	0.26	−0.08	0.18	0.3	0.26	0.32
	skewness	1.88	1.54	0.53	1.19	0.68	0.63	2.51	1.68	2.13
	coefficient or variation	55%	72%	25%	48%	38%	33%	74%	51%	69%
UR	kurtosis	−1.32	−0.68	1.1	−0.86	−0.7	−1.43	4.36	1.37	0.1
	Gini coefficient	0.35	0.17	0.1	0.19	−0.08	0.24	0.21	0.27	0.23
	skewness	0.25	0.91	−0.46	0.68	0.61	−0.06	1.84	0.92	0.87
	coefficient of variation	62%	32%	19%	34%	43%	41%	43%	50%	41%
WAP	kurtosis	2.14	−1.6	1.64	−0.62	4.06	0.29	−0.33	−0.42	−1.09
	Gini coefficient	0.01	0.01	0	0.01	0	0.01	0.01	0.01	0.01
	skewness	−0.54	0.04	1.18	0.32	1.96	−0.53	−0.12	−0.37	0.12
	coefficient or variation	1%	1%	1%	1%	1%	1%	1%	2%	2%
GDP	kurtosis	−0.42	4.02	1.89	4.05	−0.81	−0.93	0.99	5	1.63
	Gini coefficient	0.14	0.09	0.16	0.08	−0.02	0.11	0.16	0.14	0.09
	skewness	0.68	1.37	1.24	1.62	0.5	0.75	1.49	2.06	1.46
	coefficient or variation	25%	18%	29%	16%	15%	20%	33%	28%	17%
OSR	kurtosis	1.14	2.92	1.61	3.93	4.57	0.05	1.96	5.92	−0.38
	Gini coefficient	0.28	0.25	0.24	0.28	0.21	0.17	0.27	0.21	0.27
	skewness	1.13	1.52	0.9	1.74	2.08	−0.45	1.66	1.67	0.57
	coefficient or variation	53%	48%	46%	55%	45%	30%	56%	42%	48%
TG	kurtosis	−0.46	0.27	0.9	2.44	0.42	1.52	−1.13	−0.09	−0.83
	Gini coefficient	0.4	0.19	0.17	0.23	0.16	0.17	0.11	0.27	0.26
	skewness	0.77	1.08	0.77	1.19	−0.96	−0.55	−0.21	0.44	0.36
	coefficient or variation	71%	36%	32%	44%	30%	33%	20%	48%	46%
GS	kurtosis	−0.74	−0.38	−1.92	5.14	1.32	0.88	−0.37	0.17	−0.79
	Gini coefficient	0.39	0.25	0.21	0.23	0.17	0.19	0.12	0.29	0.29
	skewness	0.72	0.9	0.43	1.82	−1.18	−0.51	0.14	0.68	0.46
	coefficient or variation	70%	45%	38%	47%	33%	35%	22%	53%	51%
FR	kurtosis	0.66	−0.81	−0.28	0.65	4.46	0	4.72	0.99	−1.04
	Gini coefficient	0.04	0.05	0.07	0.03	0.05	0.02	0.04	0.05	0.06
	skewness	0.97	1.01	1.05	1.3	2.03	0.31	2.09	1.13	0.53
	coefficient or variation	8%	9%	12%	7%	11%	4%	8%	10%	10%
PPE	kurtosis	0.11	−0.35	−2.16	0.49	0.03	−0.62	−1.15	−0.47	−0.92
	Gini coefficient	0.04	0.1	0.07	0.08	0.02	0.07	0.07	0.1	0.09
	skewness	−0.84	−0.31	0.17	0.33	0.08	−0.66	0.64	−0.06	−0.54
	coefficient or variation	7%	18%	13%	15%	4%	13%	13%	18%	17%
WSS	kurtosis	0.37	0.6	−1.9	-0.59	3.5	7.97	1	5.42	−0.71
	Gini coefficient	0.13	0.01	0.04	0.02	0.02	0.07	0.03	0.07	0.04
	skewness	−1.25	−0.94	-0.84	−0.51	−1.81	−2.67	−0.83	−2.24	−0.71
	coefficient or variation	25%	3%	8%	4%	5%	17%	5%	16%	7%
SS	kurtosis	1.11	−0.96	−0.4	−0.73	1.01	−1.36	3.32	−0.88	−0.54
	Gini coefficient	0.16	0.11	0.12	0.04	0.2	0.28	0.15	0.22	0.16
	skewness	0.95	−0.21	−0.07	0.08	0.48	0.6	1.4	0.26	−0.22
	coefficient or variation	31%	20%	21%	26%	36%	50%	31%	39%	28%
GSS	kurtosis	−1.86	1.77	−0.52	2.69	−0.32	−1.45	2.43	0.95	−0.31
	Gini coefficient	0.34	0.38	0.33	−0.03	0.49	0.5	0.51	0.57	0.39
	skewness	−0.12	1.41	0.7	1.59	0.69	0.5	1.75	1.46	1.01
	coefficient of variation	61%	73%	60%	81%	90%	90%	109%	114%	74%

**Table 5:** Herfindahl–Hirschman Index (HHI) coefficient for four categories of health determinant (SDH).

Variables	PL2	PL4	PL5	PL6	PL7	PL8	PL9	PLW	PLM
FM	830	1000	1670	830	1670	910	1430	200	590
ODR	850	1000	1680	840	1670	920	1440	210	600
ER1	1100	1120	1900	930	2050	1070	1680	310	890
ER2	1130	1220	1880	930	1960	1050	1580	260	680
ER3	940	1800	2360	990	1970	990	2380	230	850
ER4	1080	1510	1770	1030	1910	1010	2210	260	870
UR	1150	1100	1720	930	1970	1060	1700	250	690
WAP	830	1000	1670	830	1670	910	1430	200	590
GDP	880	1030	1810	850	1700	940	1580	220	610
OSR	1070	1230	2020	1080	2010	990	1880	240	730
TG	1260	1130	1830	990	1820	1010	1480	250	710
GS	1240	1210	1900	1010	1840	1020	1500	260	740
FR	840	1010	1690	840	1690	910	1440	210	590
FR	840	1030	1690	850	1670	920	1450	210	600
WSS	880	1000	1680	830	1670	930	1430	210	590
SS	910	1040	1740	890	1880	1140	1560	230	630
GSS	1140	1530	2270	1380	3020	1650	3120	470	910

No shading—low concentration; 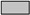
—moderate concentration; 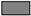
—strong concentration.

**Table 6:** The least squares (OLS) Model 1 (dependent variable LE(m) = 0).

Variable	Coefficient	Standard Error	t-Student Ratio	*p*-Value	
const	4.29715	0.189800	22.64	<0.0001	***
l_FM	0.172368	0.0401624	4.292	<0.0001	***
l_ODR	−0.0220687	0.00447879	−4.927	<0.0001	***
l_WAP	−0.234497	0.0143205	−16.37	<0.0001	***
l_OSR	−0.00778231	0.00170110	−4.575	<0.0001	***
l_TG	−0.0168314	0.00147889	−11.38	<0.0001	***
l_GS	0.0242238	0.00187487	12.92	<0.0001	***
l_PPE	0.0170003	0.00113016	15.04	<0.0001	***
l_WSS	−0.0100748	0.00305660	−3.296	0.0011	***
l_SS	0.0190487	0.00142616	13.36	<0.0001	***
l_GSS	0.00204122	0.000454434	4.492	<0.0001	***

*** significance level α = 0.01.

**Table 7:** Model 1 fitting measures (dependent variable LE(m) = 0).

Sum of the Rests’ Squares	326.0792	Standard Error of Rests	1.004755
R-square	0.849887	Adjusted R-square	0.845239
F(10, 323)	182.8707	*p*-value for F test	1.5 × 10^−126^
Log credibility	−469.9173	Akaike criterion	961.8347
Schwarz criterion	1003.757	Hannan–Quinn criterion	978.5498
Basic statistics for original data			
Average value of the dependent variable	4.272378	Standard deviation of the dependent variable	0.022951
Sum of the rests’ squares	0.047350	Standard error of rests	0.012108

**Table 8:** OLS model 2 (dependent variable LE(f) = 0).

Variable	Coefficient	Standard Error	t-Student Ratio	*p*-Value	
const	5.14969	0.120998	42.56	<0.0001	***
l_FM	−0.0865969	0.0260669	−3.322	0.0010	***
l_ODR	0.0257366	0.00296186	8.689	<0.0001	***
l_WAP	−0.0914863	0.00906290	−10.09	<0.0001	***
l_TG	−0.00544797	0.000911869	−5.975	<0.0001	***
l_GS	0.0108564	0.00106811	10.16	<0.0001	***
l_PPE	0.00644938	0.000691195	9.331	<0.0001	***
l_WSS	−0.00541621	0.00146425	−3.699	0.0003	***
l_SS	0.00452266	0.000936597	4.829	<0.0001	***
l_GSS	0.00281502	0.000255847	11.00	<0.0001	***

*** significance level α = 0.01.

**Table 9:** Model 2 fitting measures (dependent variable LE(f) = 0).

Sum of the Rests’ Squares	330.9372	Standard Error of Rests	1.010649
R-squared	0.809356	Adjusted R-squared	0.804061
F(10, 323)	152.8341	*p*-value for F test	5.8 × 10^−111^
Log credibility	−472.3870	Akaike criterion	964.7741
Schwarz criterion	1002.885	Hannan–Quinn criterion	979.9696
Basic statistics for original data			
Average value of dependent variable	4.390039	Standard deviation of dependent variable	0.013336
Sum of the rests’ squares	0.018890	Standard error of rests	0.007636
